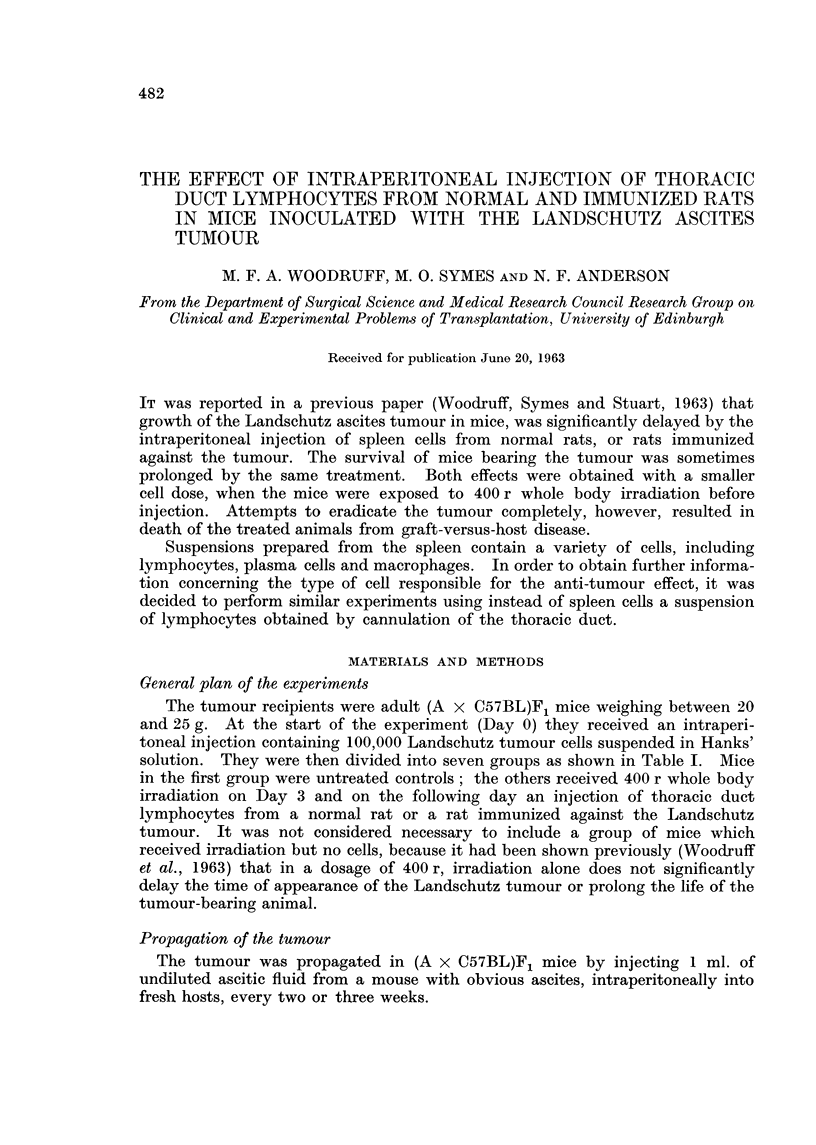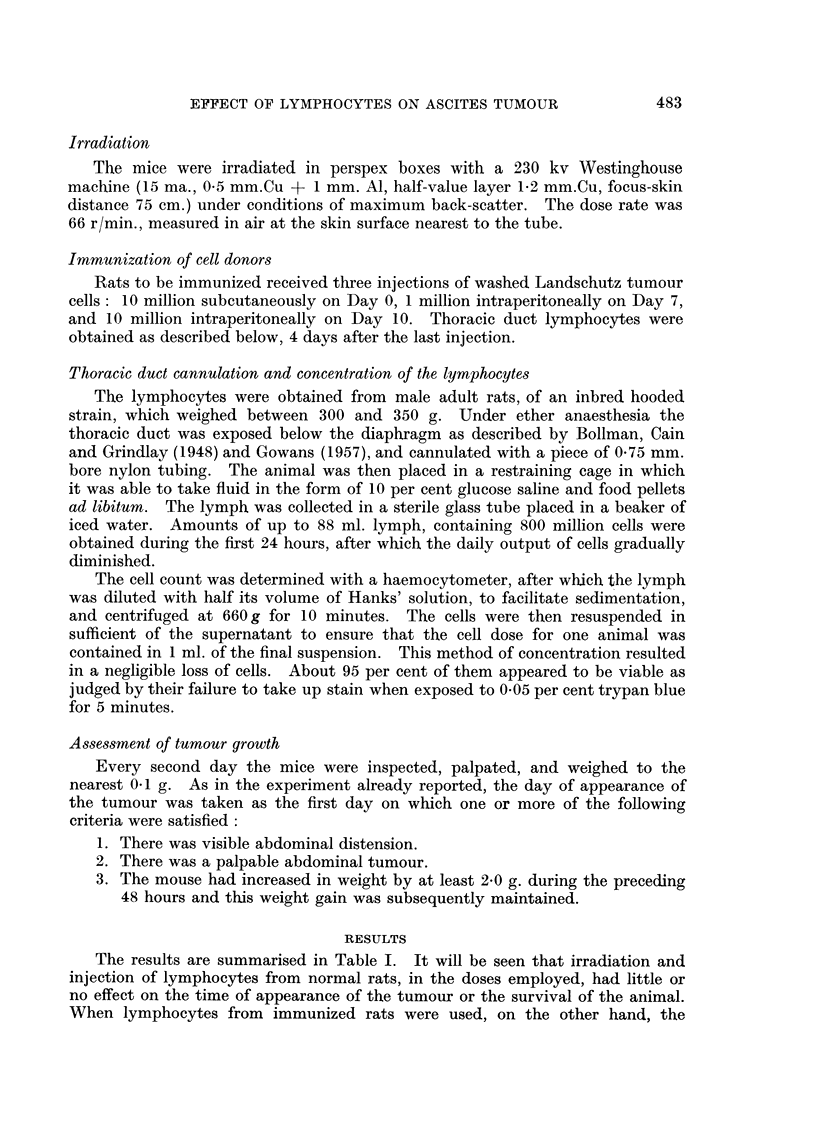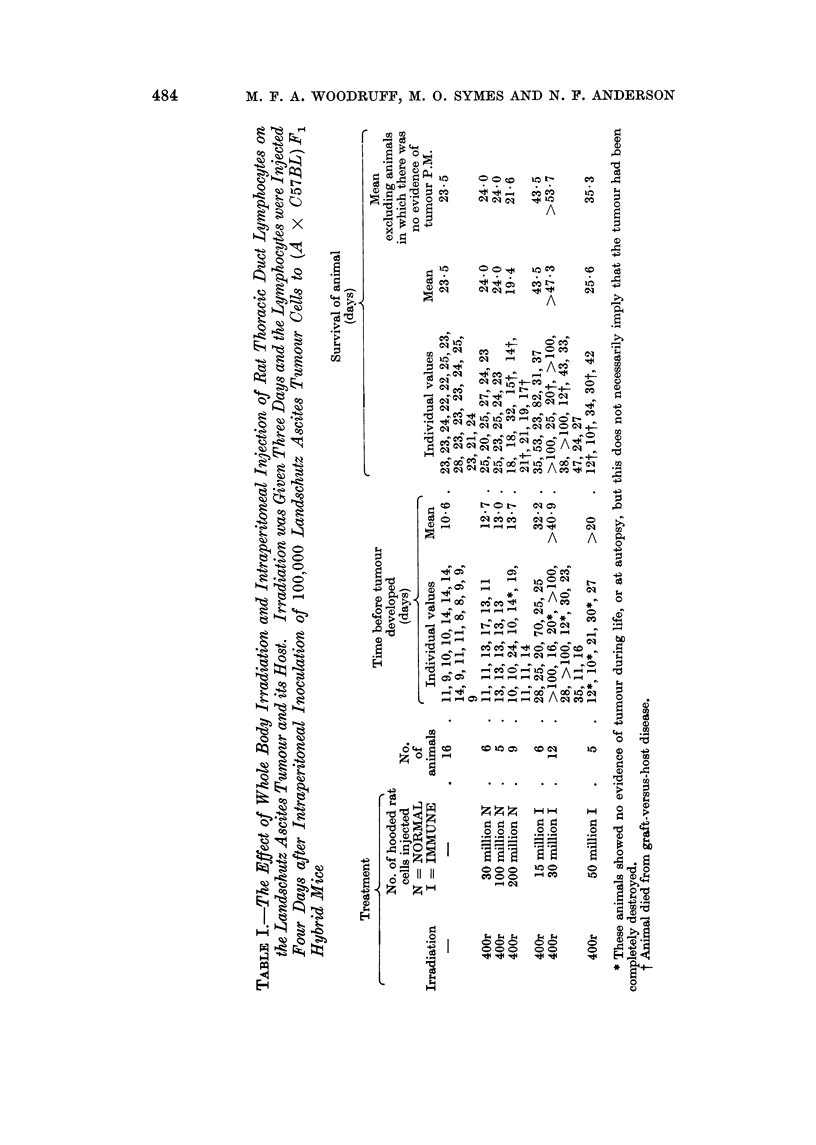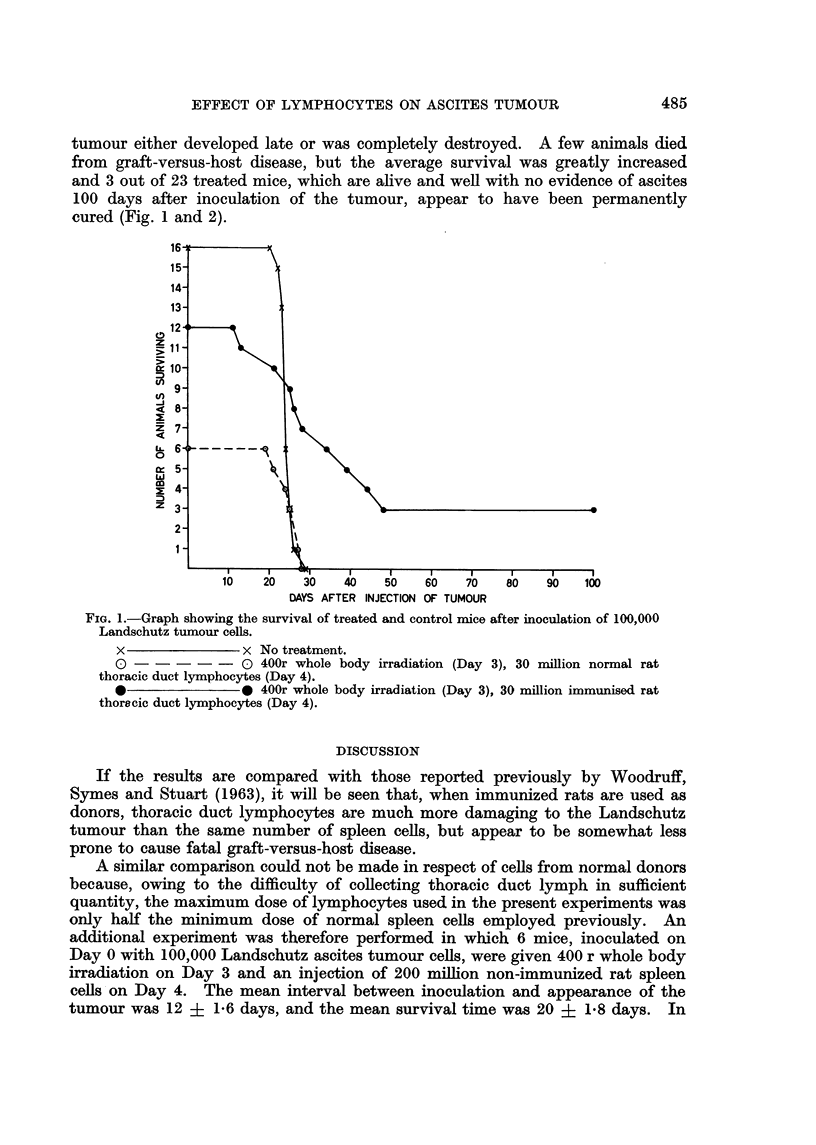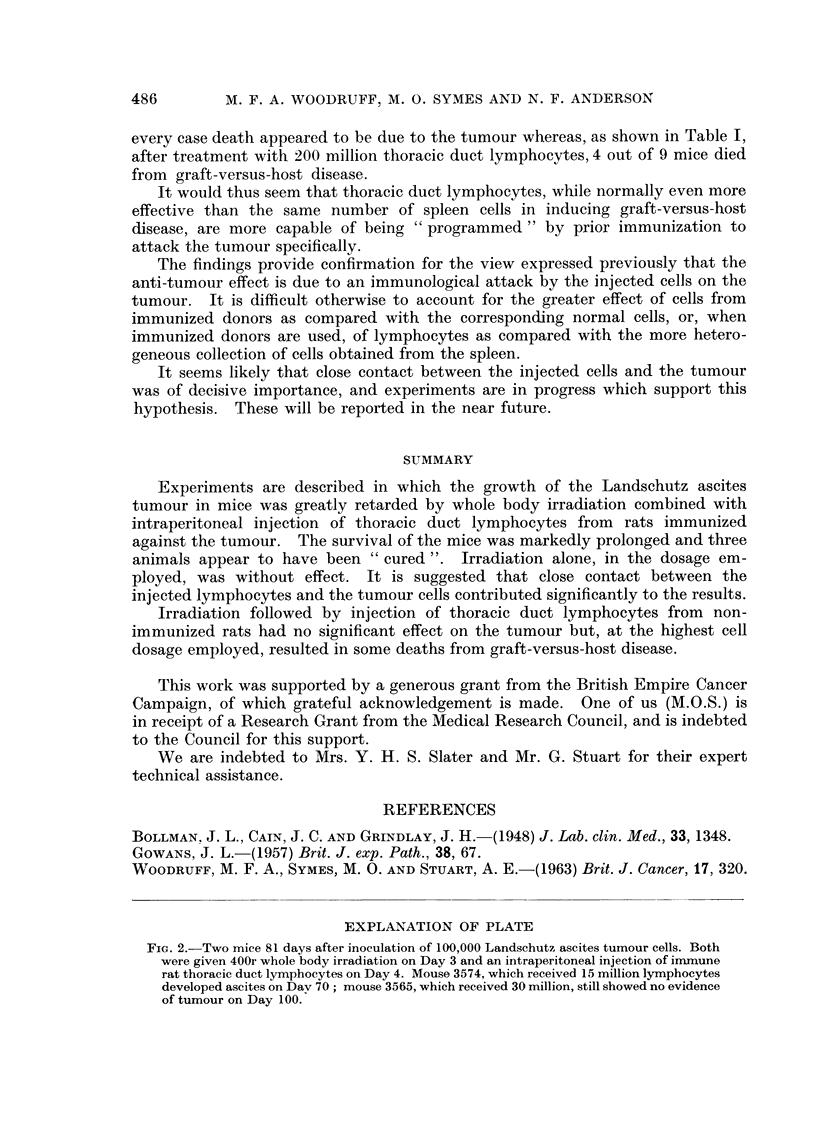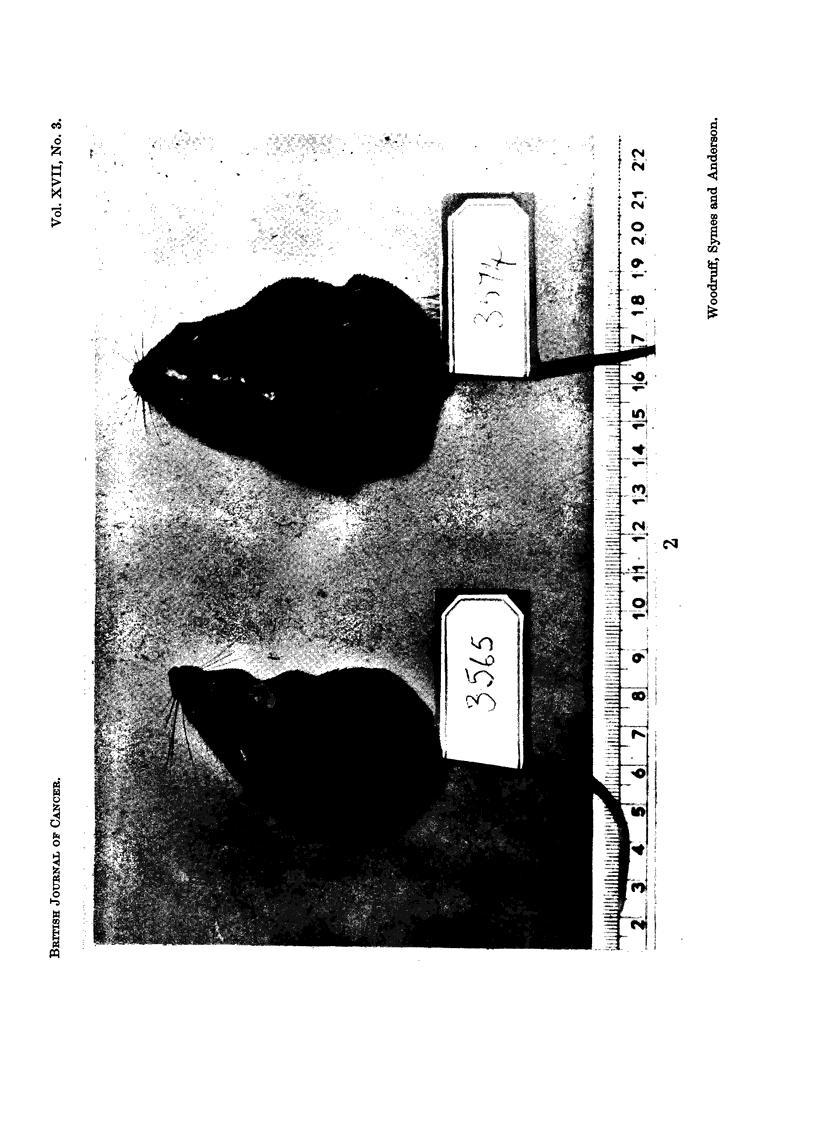# The Effect of Intraperitoneal Injection of Thoracic Duct Lymphocytes from Normal and Immunized Rats in Mice Inoculated with the Landschutz Ascites Tumour

**DOI:** 10.1038/bjc.1963.65

**Published:** 1963-09

**Authors:** M. F. A. Woodruff, M. O. Symes, N. F. Anderson

## Abstract

**Images:**


### THE EFFECT OF INTRAPERITONEAL INJECTION OF THORACIC DUCT LYMPHOCYTES FROM NORMAL AND IMMUNIZED RATS IN MICE INOCULATED WITH THE LANDSCHUTZ ASCITES TUMOUR

M. F. A. WOODRUFF, M. O. SYMES AND N. F. ANDERSON

*From the Department of Surgical Science and Medical Research Council Research Group on Clinical and Experimental Problems of Transplantation, University of Edinburgh*

Received for publication June 20, 1963

IT was reported in a previous paper (Woodruff, Symes and Stuart, 1963) that growth of the Landschutz ascites tumour in mice, was significantly delayed by the intraperitoneal injection of spleen cells from normal rats, or rats immunized against the tumour. The survival of mice bearing the tumour was sometimes prolonged by the same treatment. Both effects were obtained with a smaller cell dose, when the mice were exposed to 400 r whole body irradiation before injection. Attempts to eradicate the tumour completely, however, resulted in death of the treated animals from graft-versus-host disease.

Suspensions prepared from the spleen contain a variety of cells, including lymphocytes, plasma cells and macrophages. In order to obtain further information concerning the type of cell responsible for the anti-tumour effect, it was decided to perform similar experiments using instead of spleen cells a suspension of lymphocytes obtained by cannulation of the thoracic duct.

### MATERIALS AND METHODS

### *General plan of the experiments*

The tumour recipients were adult (A $\times$ C57BL)$F_1$ mice weighing between 20 and 25 g. At the start of the experiment (Day 0) they received an intraperitoneal injection containing 100,000 Landschutz tumour cells suspended in Hanks' solution. They were then divided into seven groups as shown in Table I. Mice in the first group were untreated controls ; the others received 400 r whole body irradiation on Day 3 and on the following day an injection of thoracic duct lymphocytes from a normal rat or a rat immunized against the Landschutz tumour. It was not considered necessary to include a group of mice which received irradiation but no cells, because it had been shown previously (Woodruff *et al.*, 1963) that in a dosage of 400 r, irradiation alone does not significantly delay the time of appearance of the Landschutz tumour or prolong the life of the tumour-bearing animal.

### *Propagation of the tumour*

The tumour was propagated in (A $\times$ C57BL)$F_1$ mice by injecting 1 ml. of undiluted ascitic fluid from a mouse with obvious ascites, intraperitoneally into fresh hosts, every two or three weeks.

*Irradiation*

The mice were irradiated in perspex boxes with a 230 kv Westinghouse machine (15 ma., 0·5 mm.Cu + 1 mm. Al, half-value layer 1·2 mm.Cu, focus-skin distance 75 cm.) under conditions of maximum back-scatter. The dose rate was 66 r/min., measured in air at the skin surface nearest to the tube.

*Immunization of cell donors*

Rats to be immunized received three injections of washed Landschutz tumour cells : 10 million subcutaneously on Day 0, 1 million intraperitoneally on Day 7, and 10 million intraperitoneally on Day 10. Thoracic duct lymphocytes were obtained as described below, 4 days after the last injection.

*Thoracic duct cannulation and concentration of the lymphocytes*

The lymphocytes were obtained from male adult rats, of an inbred hooded strain, which weighed between 300 and 350 g. Under ether anaesthesia the thoracic duct was exposed below the diaphragm as described by Bollman, Cain and Grindlay (1948) and Gowans (1957), and cannulated with a piece of 0·75 mm. bore nylon tubing. The animal was then placed in a restraining cage in which it was able to take fluid in the form of 10 per cent glucose saline and food pellets *ad libitum.* The lymph was collected in a sterile glass tube placed in a beaker of iced water. Amounts of up to 88 ml. lymph, containing 800 million cells were obtained during the first 24 hours, after which the daily output of cells gradually diminished.

The cell count was determined with a haemocytometer, after which the lymph was diluted with half its volume of Hanks' solution, to facilitate sedimentation, and centrifuged at 660 g for 10 minutes. The cells were then resuspended in sufficient of the supernatant to ensure that the cell dose for one animal was contained in 1 ml. of the final suspension. This method of concentration resulted in a negligible loss of cells. About 95 per cent of them appeared to be viable as judged by their failure to take up stain when exposed to 0·05 per cent trypan blue for 5 minutes.

*Assessment of tumour growth*

Every second day the mice were inspected, palpated, and weighed to the nearest 0·1 g. As in the experiment already reported, the day of appearance of the tumour was taken as the first day on which one or more of the following criteria were satisfied :

1. There was visible abdominal distension.
2. There was a palpable abdominal tumour.
3. The mouse had increased in weight by at least 2·0 g. during the preceding 48 hours and this weight gain was subsequently maintained.

### RESULTS

The results are summarised in Table I. It will be seen that irradiation and injection of lymphocytes from normal rats, in the doses employed, had little or no effect on the time of appearance of the tumour or the survival of the animal. When lymphocytes from immunized rats were used, on the other hand, the tumour either developed late or was completely destroyed. A few animals died from graft-versus-host disease, but the average survival was greatly increased and 3 out of 23 treated mice, which are alive and well with no evidence of ascites 100 days after inoculation of the tumour, appear to have been permanently cured (Fig. 1 and 2).

FIG. 1.—Graph showing the survival of treated and control mice after inoculation of 100,000 Landschutz tumour cells.

× ———————— × No treatment.

○ — — — — — — ○ 400r whole body irradiation (Day 3), 30 million normal rat thoracic duct lymphocytes (Day 4).

● ———————— ● 400r whole body irradiation (Day 3), 30 million immunised rat thoracic duct lymphocytes (Day 4).

### DISCUSSION

If the results are compared with those reported previously by Woodruff, Symes and Stuart (1963), it will be seen that, when immunized rats are used as donors, thoracic duct lymphocytes are much more damaging to the Landschutz tumour than the same number of spleen cells, but appear to be somewhat less prone to cause fatal graft-versus-host disease.

A similar comparison could not be made in respect of cells from normal donors because, owing to the difficulty of collecting thoracic duct lymph in sufficient quantity, the maximum dose of lymphocytes used in the present experiments was only half the minimum dose of normal spleen cells employed previously. An additional experiment was therefore performed in which 6 mice, inoculated on Day 0 with 100,000 Landschutz ascites tumour cells, were given 400 r whole body irradiation on Day 3 and an injection of 200 million non-immunized rat spleen cells on Day 4. The mean interval between inoculation and appearance of the tumour was 12 ± 1·6 days, and the mean survival time was 20 ± 1·8 days. In every case death appeared to be due to the tumour whereas, as shown in Table I, after treatment with 200 million thoracic duct lymphocytes, 4 out of 9 mice died from graft-versus-host disease.

It would thus seem that thoracic duct lymphocytes, while normally even more effective than the same number of spleen cells in inducing graft-versus-host disease, are more capable of being " programmed " by prior immunization to attack the tumour specifically.

The findings provide confirmation for the view expressed previously that the anti-tumour effect is due to an immunological attack by the injected cells on the tumour. It is difficult otherwise to account for the greater effect of cells from immunized donors as compared with the corresponding normal cells, or, when immunized donors are used, of lymphocytes as compared with the more heterogeneous collection of cells obtained from the spleen.

It seems likely that close contact between the injected cells and the tumour was of decisive importance, and experiments are in progress which support this hypothesis. These will be reported in the near future.

### SUMMARY

Experiments are described in which the growth of the Landschutz ascites tumour in mice was greatly retarded by whole body irradiation combined with intraperitoneal injection of thoracic duct lymphocytes from rats immunized against the tumour. The survival of the mice was markedly prolonged and three animals appear to have been " cured ". Irradiation alone, in the dosage employed, was without effect. It is suggested that close contact between the injected lymphocytes and the tumour cells contributed significantly to the results.

Irradiation followed by injection of thoracic duct lymphocytes from non-immunized rats had no significant effect on the tumour but, at the highest cell dosage employed, resulted in some deaths from graft-versus-host disease.

This work was supported by a generous grant from the British Empire Cancer Campaign, of which grateful acknowledgement is made. One of us (M.O.S.) is in receipt of a Research Grant from the Medical Research Council, and is indebted to the Council for this support.

We are indebted to Mrs. Y. H. S. Slater and Mr. G. Stuart for their expert technical assistance.

### REFERENCES

Bollman, J. L., Cain, J. C. and Grindlay, J. H.—(1948) *J. Lab. clin. Med.*, **33**, 1348.
Gowans, J. L.—(1957) *Brit. J. exp. Path.*, **38**, 67.
Woodruff, M. F. A., Symes, M. O. and Stuart, A. E.—(1963) *Brit. J. Cancer*, **17**, 320.

---

### EXPLANATION OF PLATE

Fig. 2.—Two mice 81 days after inoculation of 100,000 Landschutz ascites tumour cells. Both were given 400r whole body irradiation on Day 3 and an intraperitoneal injection of immune rat thoracic duct lymphocytes on Day 4. Mouse 3574, which received 15 million lymphocytes developed ascites on Day 70 ; mouse 3565, which received 30 million, still showed no evidence of tumour on Day 100.

2

Woodruff, Symes and Anderson.